# Computational Identification of Post Translational Modification Regulated RNA Binding Protein Motifs

**DOI:** 10.1371/journal.pone.0137696

**Published:** 2015-09-14

**Authors:** Andrew S. Brown, Bidyut K. Mohanty, Philip H. Howe

**Affiliations:** 1 Department of Biochemistry, Medical University of South Carolina, Charleston, South Carolina, United States of America; 2 Department of Biomedical Science, Kent State University, Kent, Ohio, United States of America; The John Curtin School of Medical Research, AUSTRALIA

## Abstract

RNA and its associated RNA binding proteins (RBPs) mitigate a diverse array of cellular functions and phenotypes. The interactions between RNA and RBPs are implicated in many roles of biochemical processing by the cell such as localization, protein translation, and RNA stability. Recent discoveries of novel mechanisms that are of significant evolutionary advantage between RBPs and RNA include the interaction of the RBP with the 3’ and 5’ untranslated region (UTR) of target mRNA. These mechanisms are shown to function through interaction of a *trans-*factor (RBP) and a *cis-*regulatory element (3’ or 5’ UTR) by the binding of a RBP to a regulatory-consensus nucleic acid motif region that is conserved throughout evolution. Through signal transduction, regulatory RBPs are able to temporarily dissociate from their target sites on mRNAs and induce translation, typically through a post-translational modification (PTM). These small, regulatory motifs located in the UTR of mRNAs are subject to a loss-of-function due to single polymorphisms or other mutations that disrupt the motif and inhibit the ability to associate into the complex with RBPs. The identification of a consensus motif for a given RBP is difficult, time consuming, and requires a significant degree of experimentation to identify each motif-containing gene on a genomic scale. We have developed a computational algorithm to analyze high-throughput genomic arrays that contain differential binding induced by a PTM for a RBP of interest–RBP-PTM Target Scan (RPTS). We demonstrate the ability of this application to accurately predict a PTM-specific binding motif to an RBP that has no antibody capable of distinguishing the PTM of interest, negating the use of *in-vitro* exonuclease digestion techniques.

## Introduction

Post-translational modification (PTM) of RBPs through signal transduction can have tremendous implications for affinity to conserved RNA sequences in the 3’ and 5’ UTRs [1]. Signal transduction cascades cause various PTMs of RBPs that have the potential to effect stability, localization, conformation, and affinity to RNA [2, 3]. This study aims to predict the nucleic acid regulatory motif to which a specific RBP’s affinity is modulated through PTM.

Cellular regulation through conserved regulatory motifs in UTR regions of mRNA is an essential process that maintains homeostasis. RBPs interact with motifs in mRNA and control its stability, translation, and localization; all of which play a vital role in the progression of disease [4]. Advances in high-throughput, genome wide sequencing have allowed for genomic RBP analysis by cost-effective, *in-vitro* binding assays coupled to downstream sequencing. A need has been established for computational tools to aid with the analysis of data generated in these studies to give accurate predictions of regulatory nucleic acid target sites that bind RBPs in a PTM-dependent manner.

The conservation of regulatory motifs in orthologous genes across evolution is a guiding principle in the analysis of high-throughput sequencing data generated from RBP binding studies [5–7]. 3’ and 5’-UTRs contain conserved motifs that interact with RBPs, these motifs are conserved, while the overall UTR has become divergent in both sequence and length. These divergent species have shown similar regulatory activity despite their highly variable UTRs [8]. To illustrate this finding, a complex of RBPs and a 3’-UTR motif are shown to regulate the expression of genes responsible for inflammation that is induced by stimulation with interferon (IFN)-γ. IFN-γ was shown to silence the expression of these inflammatory mediators upon PTM to RBPS involved in forming a complex with a conserved 3’-UTR element. The modification of these RBPs facilitated their dissociation from their target nucleic acid motif and abolished the inhibitory effect of complex formation. *In-vitro* studies were performed using a tedious method of sequentially shortening the 3’-UTR and determining binding affinity for the RBP. When the minimal sequence was found it was compared for evolutionary conservation and found to be highly conserved in the 3’-UTR of orthologous genes that had a highly variable 3’ UTR length when compared to one another [9].

Databases such as Entrez hosted on PubMed (ncbi.nlm.nih.gov) allow for the fast and efficient retrieval of nucleic acid and protein sequences, as well as experimental results that contain data from high-throughput array studies [10]. The GEO database contains many studies where the genomic affinity of 3’ and 5’-UTRs are characterized as a function of modifying an RBP. Many of the conclusions drawn from these studies simply include the signature of gene induction that was derived from analyzing the transcriptome expression levels for each sample. The underlying mechanism of these genetic alterations lies in the interaction between an RBP and a consensus nucleic acid motif that is contained in the genes directly interacting with the RBP. We have developed an algorithm capable of analyzing array data and through evolutionary analysis determine a minimal regulatory-consensus motif descriptor that has affinity for an individual RBP as a function of PTM.

There are many methods that utilize a lengthy process involving exonuclease digestion of RBP-bound RNA, coupled to primer ligation and downstream high-throughput sequencing. The intention of this algorithm is to detect a regulatory motif that is sensitive to PTM of a specific RBP, this could include the loss, or gain of binding induced by the PTM. In a situation where there are no commercially available antibodies capable of distinguishing a PTMs from native RBPs, this application serves as an excellent tool to predict motifs that can be later validated by multiple *in-vitro* binding assays.

The algorithm presented in this work has been tested on a publicly available 3’ UTR Affymetrix array (GEO, GSE40466) that was used to determine the genome wide loss-of-binding of the RBP hnRNP E1 as a function of TGFβ induced post-translation modification. hnRNP E1 is a protein that is implicated in the translational silencing of genes through binding a *cis-*regulatory element in the 3’ UTR of mature mRNA. The complex formed by hnRNP E1 and the 3’ UTR regulatory element can be disrupted by the phosphorylation of hnRNP E1^Ser43^ (p-hnRNP E1) induced by TGFβ [11, 12]. This analysis identifies 36 genes that have a significant loss-of-binding to p-hnRNP E1 [13]. We demonstrate the ability of our algorithm to predict a consensus motif and support this prediction with *in-vitro* analysis of binding kinetics.

## Materials and Methods

### RPTS Package

Here we introduce RNA Binding protein Post-translational modification Regulatory Nucleic Acid Target Predictor (RPTS), a computational analysis tool implemented in python, capable of identifying putative nucleic acid consensus motifs that interact with a specific RBP in a PTM-dependent manner. We have made available a repository located at: https://github.com/asbrown001/RPTS, containing source code. Installation and configuration instructions are located in the root directory. This tool is a high-throughput wrapper that builds off of the Clustal-Ω alignment engine and Bioconductor statistical computing package (implemented in R). We handle pre-processing of raw high-throughput data with optional libraries that are user selectable. Table 1 provides a detailed description of the libraries that are selectable to perform pre-processing operations on raw data. To minimize configuration issues, certain optional libraries are not able to be run locally on the client machine, we have made available webservices that utilize a preconfigured server to handle pre-processing and return a fully processed Javascript object notation (JSON) object. We detect differential expression of processed gene expression data by utilizing the limma package for Bioconductor, and handle multiple *p-value* correction using the Bonferroni correction method, executed by R. This application executes Bioconductor / R scripts by utilizing the RPy2 library (http://rpy.sourceforge.net/), and built-in process module to execute C/C++ libraries used in alignment.

**Table 1:** Optional Bioconductor chip reading libraries.

Library	Chip Type	Run Local?
affy	Affymetrix 3'-biased	Yes
gcrma	Affymetrix 3'-biased	Yes
affyPLM	Affymetrix 3'-biased	Yes
xps	Affymetrix 3'-biased	No
oligo	Affymetrix Exon ST	Yes
exonmap	Affymetrix Exon ST	No
xps	Affymetrix Exon ST	No
oligo	Affymetrix Gene ST	Yes
xps	Affymetrix Gene ST	No
oligo	Affymetrix SNP	Yes
oligo	Affymetrix Tilling	Yes
oligo	Nimblegen	Yes
lumi	Illumina	No
beadarray	Illumina	No

### Computational Algorithm RPTS

The analysis used in this publication uses a publicly available dataset hosted in GEO, GSE40466 that contain data from 36 unique Affymetrix 3’ UTR chips. The study can be downloaded as a tape archive (.tar) file that contains 36 g-zipped (.gz) files corresponding to an individual chip. RPTS allows the.tar file to be imported as input and handles all subsequent compression, decompression and computer memory management.

RPTS handles normalization of raw data and allows a user to specify experimental groups with separate samples. Statistical analysis for differentially expressed genes is carried out by the limma Bioconductor package, multiple *p-value* correction is carried out using the Bonferroni correction method (executed by R). Phenotype data that describes the experimental design can be input as a file or user-defined by entering data at prompts. Robust multichip average (RMA) normalization is applied to the processed data, fit to experimental design, and an empirical Bayes adjustment is performed to determine differentially expressed genes. Genes that had significant differential expression between different sample groups (PTM treatments) are listed to the user along with the degree of enrichment. Differentially expressed genes are next subjected to evolutionary analysis for conserved motif regions.

### Identification of Evolutionary Conserved Motifs

The list of genes found to be significantly altered by experimental treatment is further scrutinized for evolutionary conservation. RPTS contains 3’ and 5’-UTR databases from UTRdb [14, 15] for a variety of evolutionary divergent species. Key factors for choosing species, along with various programmatic options are described above.

RPTS contains a post-alignment algorithm that is used to compare evolutionarily conserved motifs. A recursive process to choose the highest conserved region of an alignment is utilized and the consensus from this region is added to a pool of motifs from genes that were predicted to contain the regulatory-motif.

### Creating, Modifying, or Updating Query Database

This algorithm requires a database that contains nucleic acid sequences in either FASTA or UTRdb flat database format. We suggest downloading database files from UTRdb (http://utrdb.ba.itb.cnr.it/) and adding to the “Database” directory contained in the application root. This application walks the entire directory and scans for database extension or compressed files. It is suggested the user separate database files downloaded in a taxonomy based directory to allow for simple updating. The evolutionary analysis step allows a database file to be read into memory as a custom object that supports rapid querying, to update any of these files, simply modify the contents of the “Database” directory. Any modification to files in this directory will take effect on the next subsequent instantiation of the application, no further input from the user is necessary.

### Evolutionary Divergent Orthologous RBP PTM Validation

The selection of organisms used in evolutionary alignments is performed prior to evolutionary analysis. Organisms are first validated to contain an orthologous RBP, this is accomplished through performing a BLASTp query of the RBP-of-interest (RBPi). The user provides the accession number to their RBP of interest and this is used in a webservices call to the BLASTp url-endpoint. Results of BLAST analysis are interpreted by the application, by default, the threshold to consider the organism as having an orthologous RBP is > 60% sequence identity and > 80% alignment length, and an e-score of 1e^-10^ or greater. To assess the likelihood of this RBP undergoing a similar PTM, a user will provide a numerical position for the amino acid residue or range of residues that the PTM occurs on their RBPi. A Clustal alignment is performed on the orthologous RBP and RBPi, the residue range specified by the user is compared to see if there are identical amino acids or amino acids that have similar properties to the RBPi. If this test is passed, the organism is subjected to cytokine response analysis.

### Evolutionary Divergent Organism Cytokine Validation

Many PTMs are induced by cytokine stimulation, to account for this phenomenon in evolutionary analysis, we have made an optional check available to the user. If a user believes that the only to cause a PTM on their RBPi is through cytokine stimulation, they can set this constraint to choose only organisms that contain an orthologous cytokine, with user-adjustable constraints for similarity. To perform this check, a call to BLASTp is made using the RBPi accession number input by the user. By default, > 60% sequence identity, > 80% alignment length, and an e-score of 1e^-10^ or greater is used as a cutoff to consider the organism as having similar cytokine. If no orthologs are found, the organism is left out of evolutionary analysis.

### Selection of Putative RNA Motifs from UTRs

To identify putative consensus motifs based on an evolutionary conserved principal, we create objects in memory for each putative motif-containing gene. Each of these objects contains a UTR sequence of the gene contained on the high-throughput chip, and a variable number of UTR sequences from orthologous genes of evolutionary divergent organisms that fit exclusion criteria. This object is parsed into a memory stream and aligned using the Clustal-Ω alignment engine. Alignments are performed locally on a client machine for performance enhancement, this keeps from making an excess amount of web requests. Upon completion of the alignment, the output is extracted through the Python process module and analyzed for conservation. We have employed a recursive method that scans the overall alignment sequence and identifies regions of similarity based on a number of gaps. A user may define the sensitivity of region determination by adjusting the number of consecutive gaps and matches that will define a region, default values are > 6 consecutive gaps, and > 10 similar residues. Homologous regions are parsed into objects that contain portions of the alignment that correspond to the regional boundary and are given a score of conservation based on the number of identical and similar residues found in the sub-alignment. The region with highest conservation score is then selected at the putative motif for that gene and added to a collection of confirmed genes.

### Western Blot Analysis and Immunoprecipitation

To measure levels of p-hnRNP E1, we employed a combinational approach through immunoprecipitation and subsequent western blot analysis. No antibody yet exists to specifically detect p-hnRNP E1; however, there are antibodies capable of recognizing the phosphorylated form of the consensus RXRXXpS, which is contained at the Ser43 site of hnRNP E1. For immunoprecipitation, protein-A coupled sepharose was incubated with α-hnRNP E1 at 4°C for a minimum of 1 hour. Protein-A / antibody mixture was washed 3x with wash buffer (PBS, 0.05% Tween-20), and added to 500 μg total cellular lysate from NMuMG cells and incubated at 4°C for a minimum of 2 hours. Beads were washed 3x with wash buffer and loaded into polyacrylamide gel for electrophoresis, subsequently transferred to membrane for western blot analysis.

### RNA Immunoprecipitation Assay and Polymerase Chain Reaction

Antibody and protein-A coupled sepharose was prepared as described in western blot and immunoprecipitation methods above. For RNA immunoprecipitation, antibody and bead mixture was incubated with cellular lysate for a minimum of 20 minutes at 4°C. Beads were washed 3x with an RNA wash buffer (200 mM NaCl, 0.05% Tween-20), and RNA was extracted using phenol: chloroform extraction. RNA was reverse transcribed into cDNA and amplified using polymerase chain reaction, samples were electrophoresed on a 2% agarose-gel.

## Results

### Determination of RNA Consensus Binding Sequences for RNA Binding Proteins

Our example analysis of GSE40466 (GEO database) shows a number of genes that have a signature hnRNP E1 interaction that is inhibited by TGFβ induced PTM. Again, TGFβ induces p-hnRNP E1, and is shown to cause the dissociation from its target nucleic acid sequence. This novel regulatory system reported previously by our lab was shown to promote stalling at the elongation stage of translation through the interaction of hnRNP E1, a target nucleic acid sequence in the 3’-UTR, and various other proteins factors [11, 12]. hnRNP E1 was shown to be the most crucial member of this stall-complex by the observation of polyribosomal sedimentation assays. We observed samples untreated with TGFβ had enriched 80S factions (monosomal) for genes that were known to contain the nucleic acid regulatory sequence. Samples that had hnRNP E1 silenced, and samples that were treated with TGFβ saw these same genes shift into the polyribosomal fractions, indicating the necessity for intact hnRNP E1. GSE40466 contains 2 distinct experiments used to characterize genes this signature between motif containing 3’-UTRs and hnRNP E1, the first group compares differential binding to hnRNP E1 as a function of treatment with TGFβ. The second group of experiments compares enrichment of polyribosomal fractions as a function of both TGFβ treatment, and the loss of hnRNP E1. RPTS allows for these experimental groups to be defined. Experiment group 1 was set to scan for genes that had a strong affinity for hnRNP E1 and a subsequent loss with TGFβ treatment. Experimental group 2 was set to look for genes that were highly enriched in the 80S and lighter (monosomal) factions in hnRNP E1-competent cells, and a subsequent shift of enrichment into the polyribosomal fractions as either a function of hnRNP E1 silencing, or TGFβ treatment.

### RPTS Algorithm

**Fig 1:**
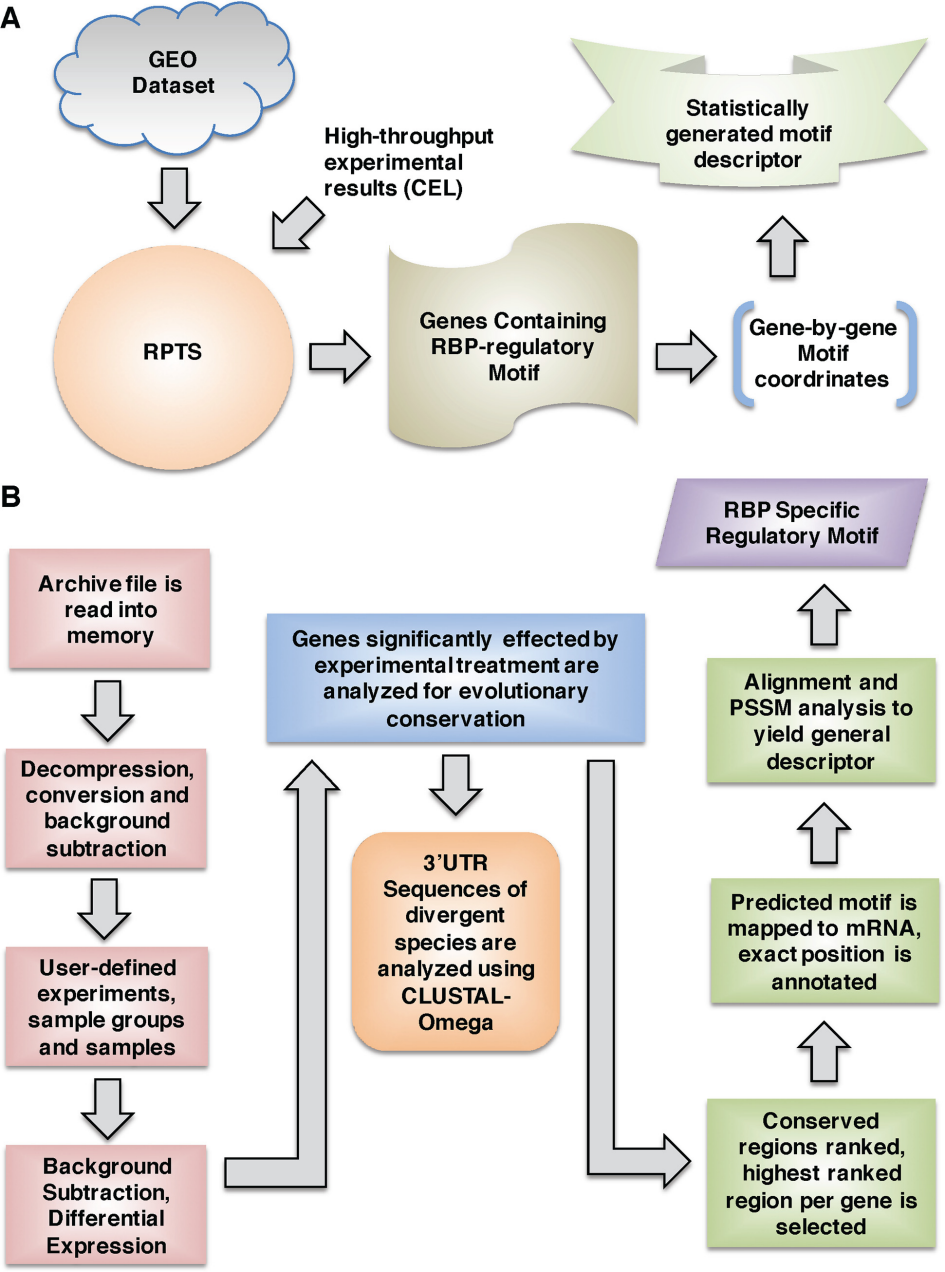
Systems overview of RPTS Application Capabilities. **A)** System overview of the user interface with RPTS. Input may come from either a published tape archive (.tar) GEO dataset, or directly from a high-throughput sequencing system, such as Affymetrix. Input data may come from multiple sources, RPTS has options the user may set in order to combine data from multiple sources. **B)** The algorithm of RPTS is described in detail in this panel. General flow of decision points and data analysis are described, key points of decision are highlighted in red-boxes. Output from the program is highlighted in green-boxes, with a description of what is contained.

We have included a diagrammatic depiction of the generalized flow-through of user interaction with RPTS to illustrate the major points of functionality of the program (**Fig 1A**). We have also included a detailed visual representation of the RPTS algorithm (**Fig 1B**). To being analyzing an experimental set of GEO data, a user may input an archive directly from GEO (http://www.ncbi.nlm.nih.gov/gds), or from a compressed version of their proprietary sequencing results. RPTS analyzes these data and gives a list of putative genes that contain the regulatory motif. The motif coordinates are given and mapped with respect to their position in mature mRNA. Each gene that contains a motif is subjected to evolutionary conservation analysis to determine the likelihood of being an RBP regulatory motif, and if validated is placed into a pool of validated genes. At the end of evolutionary analysis, all validated genes are subjected to a further alignment and a descriptor sequence is generated by analyzing the consensus of the alignment and applying a nucleic acid base generalization technique outlined in **S1 Fig.**

Once a user has loaded their datasets to RPTS, they are immediately decompressed and stored in memory. RPTS does not currently have a minimal required amount of physical Random Access Memory (RAM); however, 2 gigabytes (GB) is recommended for purposes of efficiency. Once this decompression is complete, background subtraction processing occurs utilizing the Bioconductor / R statistical computing language. Raw values of gene intensities are stored in memory, and the user then defines experimental groups that consist of individual samples (replicates) and sample groups (treatment). RPTS then determines genes that are statistically significant by normalization of replicate data, and comparison of gene expression between sample groups contained in the experiment. To accomplish statistical analysis, multiple Bioconductor packages have been made available (Table 1) depending on the type of chip used in analysis. RPTS autonomously runs Bioconductor analysis per user-specified parameters to reveal which sample groups contained significant differential expression of individual genes. Gene patters are presented to the user, they may then select the expression pattern that correlates to their expected results and proceed with evolutionary conservation analysis.

Evolutionary conservation analysis is determined by selecting the 3’-UTRs of orthologous genes of evolutionarily divergent organisms. RPTS contains compressed databases of a variety of organisms for their 3’ and 5’ UTR genomes. The conservation analysis algorithm ensures each organism selected for comparison meets the following criteria: 1) the organism must contain an orthologous RBP that is highly similar to the RBP of interest. 2) (Optional) The RBP must have the ability to undergo a similar PTM, and 3) (Optional) The organism must be capable of responding to an orthologous cytokine that is used in the experiment. Clustal-Ω alignment is performed on the orthologous genes of species that passed our algorithm’s logic. Highly conserved regions are determined and scored by a recursive algorithm, described in our experimental procedures, the highest scored region is placed into another algorithm to determine a minimal descriptor sequence based off the motif-containing genes. Finally, a descriptor sequence is generated following logic presented in the methods section. This can be used to determine critical points of interaction with the RBP, and validated using synthetic RNA expression techniques and *in-vitro* binding assays.

### Evolutionary Conservation of 3’ UTR Regulatory Motifs

**Fig 2:**
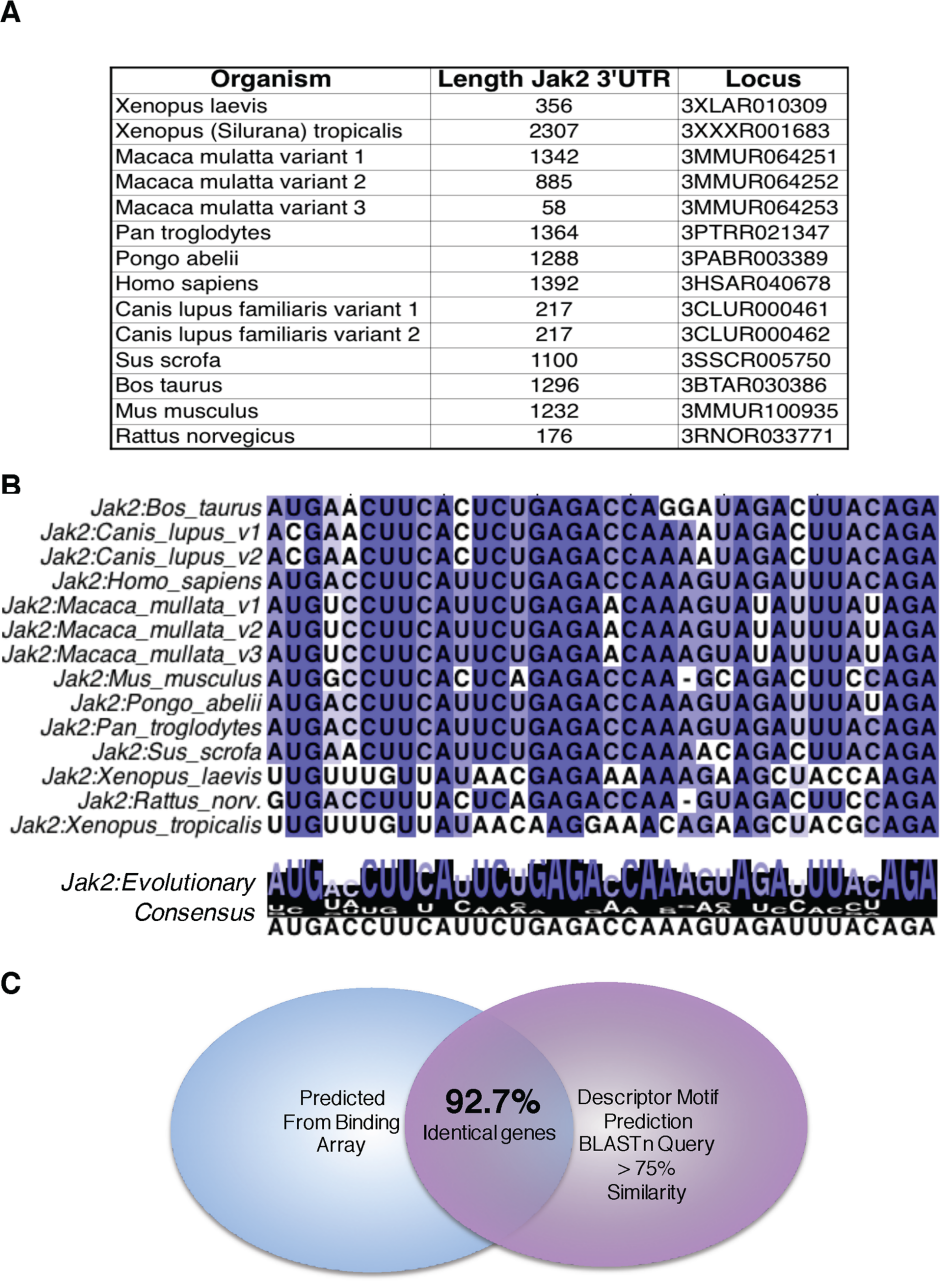
3’-UTRs become divergent across evolution while conserving regulatory motifs. **A)** Representation of the lengths of divergent species 3’-UTR Jak2 lengths. There is a significant degree of variation across evolution, however as seen in panel **B** regions of these UTRs have been conserved. **B)** This alignment was generated from RPTS, it utilizes the multiple sequence comparison by log-expectation (Clustal-Ω) alignment method to determine regions of highest conservation in Jak2 [16–18]. Purines and Pyrmidines are differentiated by pink and blue, respectively. **C)** Our training GEO set *GSE40466* yielded a consensus nucleic acid sequence specific to binding hnRNP E1, in addition to determining a consensus sequence, the output includes the identity of genes that contain this motif. When the consensus sequence was analyzed by BLASTn, 92.7% of the genes predicted by BLAST analysis were also predicted by RPTS.

We demonstrate the techniques and theory used to find probable RBP regulatory-consensus motifs from high throughput binding data. As noted earlier, the 3’ UTRs of evolutionary divergent species vary significantly in both sequence and length; therefore, an evolutionarily important regulatory sequence would likely be conserved to maintain regulation and mechanistic function. **Fig 2A** shows the divergent lengths of 3’ UTRs across Jak2, a gene regulated by the RBP of interest (hnRNP E1) in dataset GSE40466 [16–18]. The lengths of Jak2 are highly divergent; however, when aligned to the binding consensus identified by RPTS, we see this motif highly conserved across orthologous genes in the divergent species. **Fig 2B** is a Clustal-Ω [19, 20] alignment of the 3’ UTRs and the predicted RBP consensus sequence; notice the highly conserved residues in the alignment. The overall nucleic acid consensus motif that is determined by RPTS for a given dataset is compared on the transcriptome level using the basic local alignment search tool (BLAST) [21]. Given the premise that RPTS has identified a unique motif contained in a subset of genes, the descriptor motif prediction should be unique enough to use reverse-logic query genes with regions of high similarity to the motif descriptor. When analyzed to the transcriptome should yield similar genes that were predicted to contain the motif by RPTS. To confirm the regulatory-consensus motif predictions, we set out to measure the uniqueness of the motif sequence by using the basic local alignment sequence tool (BLAST). **Fig 2C** shows the overlap of BLAST-nucleotide (BLASTn) predictions obtained by analyzing the predicted consensus motif as a search pattern in the RefSeq database of *Mus musculus*. BLASTn output includes genes that contain a sequence that matches the reference pattern, genes with significant matching values were compared to the motif-containing gene predictions from RPTS. With our training set, we see a significant (92.7%) overlap of predictions, further confirming our algorithm as a robust identifier of RBP PTM regulatory binding motifs.

### Identification of Target mRNA for RNA Binding Proteins

The identification of a PTM regulatory-consensus nucleic acid binding motif for an RBP of interest is obtained by analyzing genomic wide binding arrays where gene intensity is read as a function of interaction with a RBP. This algorithm takes an approach of utilizing currently available analytic packages such as Bioconductor for detecting differentially expressed genes, and Clustal-Ω to perform alignments. It provides its own unique analytic approach to combine these tools into a high-throughput analytic package that is capable of detecting conserved regions in nucleic acid sequences. Ultimately, this application is capable of defining a region of nucleic acid responsible for binding to a RBP of interest in a PTM-dependent manner.

**Fig 3:**
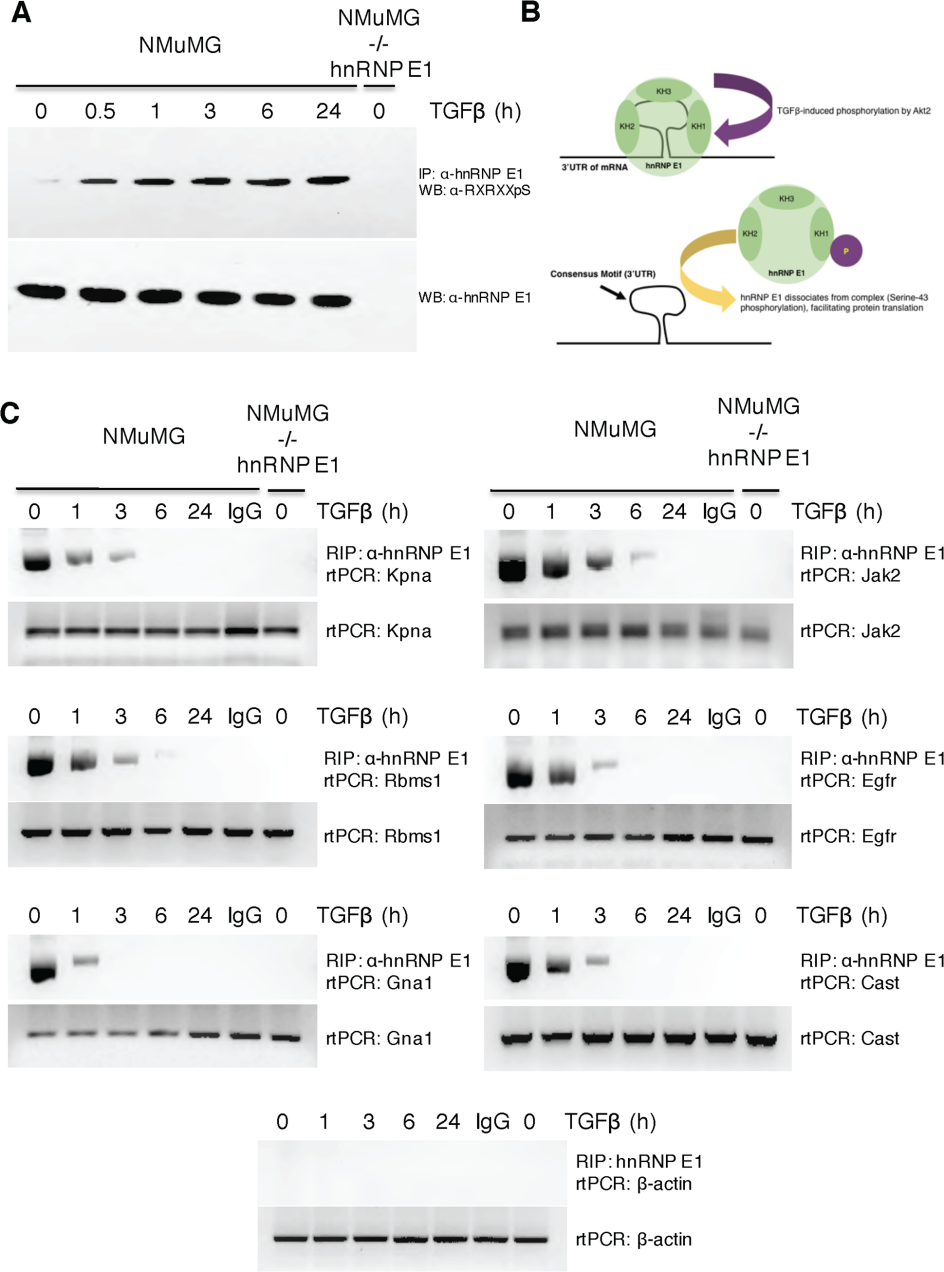
RPTS predicted motif containing genes interact with hnRNP E1. **A)** To assess the TGFβ induced serine-43 phosphorylation of hnRNP E1, we combined an immunoprecipitation and subsequent western blot analysis. Cytosolic hnRNP E1 was immunoprecipitated by α-hnRNP E1 / protein-A-sepharose, an antibody specific to the phosphorylated form of the phosphorylation consensus sequence that contains serine-43 of hnRNP E1 was used in a subsequent western blot to assay p-Ser43-hnRNP E1. NMuMG-/- hnRNP E1 samples were used as a negative control due to its lack of hnRNP E1. A steady rise of p-Ser43-hnRNP E1 is seen as NMuMG cells are treated with TGFβ in a time dependent manner. Levels of total hnRNP E1 remain unchanged as an effect of TGFβ. **B)** Schematic representation of TGFβ stimulation on hnRNP E1. TGFβ induces kinase activity of Akt2 to cause phosphorylation on serine-43 of hnRNP E1, causing dissociation of this RBP from its target consensus motif. **C)** After determining the kinetics of hnRNP E1 phosphorylation, we utilized these time points to assess and confirm the binding of RPTS predicted motif-containing genes. The top panel of individual gene assays shows the loss of binding through RNA immunoprecipitation (RIP) and subsequent reverse transcriptase polymerase chain reaction (rtPCR). We utilized IgG as a negative control to show the specificity for RIP samples with α-hnRNP E1. As seen in panel **A**, the phosphorylation levels of serine-43 peak around 3 hours, RIP analysis shows a significant loss of binding for these motif-containing genes in a direct relationship to the hnRNP E1 phosphorylation kinetics. The lower panel of gene assays shows total RNA levels as a function of TGFβ by rtPCR of total cellular RNA.

To further support our computational findings, we utilized a modified RNA-immunoprecipitation (RIP) assay to confirm the interaction (and subsequent loss of interaction through cytokine treatment) of our example RBP, hnRNP E1 with predicted motif-containing genes. NMuMG cells have been previously confirmed to actively accumulate p-hnRNP E1 in response to TGFβ signaling [11–13]. **Fig 3A** confirms the TGFβ-induced p-hnRNP E1 accumulation at various time points, keep in mind, the system demonstrated here will show a loss of interaction between motif-containing genes and p-hnRNP E1. **Fig 3B** confirms 6 predicted motif-containing genes indeed interact with our RBP of interest in a treatment dependent manner. The loss of interaction between hnRNP E1 and target genes as a function of TGFβ treatment is illustrated in **Fig 3B**. To show the effect TGFβ had on overall mRNA levels of these target genes, we assayed overall levels of the target genes mRNA from cellular lysate. As the bottom panel of each gene assay contained in **Fig 3B** shows, TGFβ does not cause a change in the total mRNA levels of these motif-containing genes.

### Contribution of Motif-Containing Genes to Various Annotated Pathways

**Fig 4:**
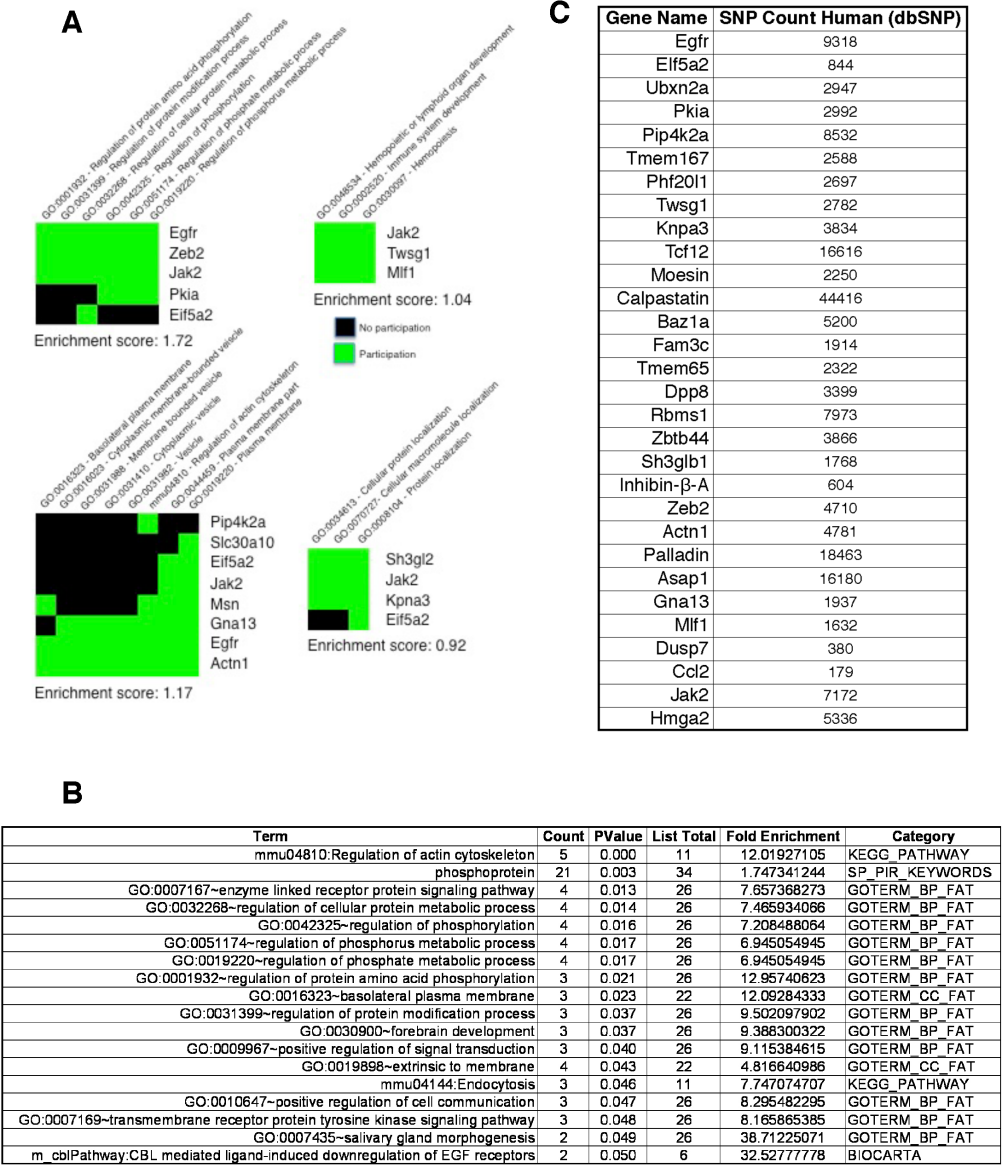
Predicted motif containing genes global set enrichment analysis. **A)** The list of motif-containing genes predicted by RPTS was analyzed by GSEA for pathway enrichment of known oncogenic pathways. Each individual block is a functional grouping of pathways, individual pathways are noted horizontally, gene names are noted vertically. **B)** GSEA individual pathway enrichment analysis. Each pathway had a *p-value* of less than 0.05, and an enrichment score of greater than 1.7-fold enrichment. When analyzed as a total group, many of these pathways are implicated in the progression of EMT, cancer, and metastasis. **C)** Previous data suggests the mutation of single bases in RBP consensus motifs causes a disruption of the complex formation. This panel shows the number of SNP records contained in dbSNP (Entrez) for each RPTS motif-containing prediction. There are significant numbers of SNP mutations that occur in the loci of these genes, it is statistically plausible that some of these mutations occur within the boundaries of individual RBP consensus motif.

Our conformational findings illustrated in **Figs 2 and 3**allow for further downstream analysis. Predictions of RPTS essentially define an RBP-mediated regulon with individual genes identified and their putative RBP-binding consensus motif precisely mapped. Our aim is to visualize a system that is regulated by an individual RBP or its PTM in the context of a biological system. To determine the contributions these regulon genes have to metabolism and known cell regulatory pathways, we utilized the Global Set Enrichment Analysis (GSEA) to analyze the contributions and metabolic pathways that would be activated by the induction of our regulon genes [22, 23]. **Fig 4A** is a representation of grouped pathways that are known to have a synergistic contribution. Analyzing our predicted regulon genes for contribution to grouped metabolic pathways, we see four groupings that are highly implicated in the progression of cancer. These findings support previous observations that TGFβ-induced pSer43 of hnRNP E1 drives both cancer progression and metastasis [24–26].

Individual metabolic pathways induced by malignancy associated with our RBP-regulon system were also determined by using RPTS. **Fig 4C** contains individual metabolic pathways that would be activated as a function of expression of our regulon genes. Again, these findings support literature that associates the loss of repressor hnRNP E1 or its PTM by TGFβ as a potent inducer of cancer. **Fig 4C** highlights pathways that contained a *p*-value of less than 0.05, and a significant (greater than 1.7) fold enrichment, many of these pathways are involved in the induction of cancer, and support the many observations that hnRNP E1 dysregulation induces cancer.

## Discussion

The development of RPTS was motivated by the need to determine a nucleic acid consensus motif specific to an RBP, and subsequently identify on a genome scale, all genes containing this regulatory motif in their UTR. The analysis presented in this manuscript benefits from the vast amounts of publicly available data to support our computational predictions and *in-vitro* observations. RPTS predictions can serve as an initial screen for analyzing high-throughput UTR assays as a function of RBP binding. The output of RPTS is directly testable through relatively inexpensive *in-vitro* methods that involve the synthetic production of both the RBP and multiple nucleic acid transcripts.

RPTS was engineered to analyze 3’ and 5’ UTR array data; however, its theory and core foundation should provide a robust platform for modification and analysis of DNA-binding protein and DNA arrays. A potential shortcoming of RPTS lies in its strict analysis algorithm where a putative gene that shows proper binding characteristics could be rejected due to a lack of evolutionary conservation in its 3’ UTR. We have justified this shortcoming by the notion that genes critical to cellular stability would have likely conserved their regulatory motifs. However, genes which have binding characteristics, yet lack a conserved motif likely have a less important role in cellular function.

Our lab has employed RPTS to thoroughly analyze and characterize a nucleic acid binding motif that is unique to the Ser43 site on hnRNP E1 in an ongoing study. This study is near completion and has vigorous *in-vitro* binding data to support RPTS predictions. We further characterize biological significance, and show a clinical consequence for the disruption of the binding between an RBP and a regulatory motif contained in an oncogenic subset of genes. With the planned additions to extend the capabilities of RPTS, we anticipate this becoming a continuously refined algorithm that gains robustness through usage by other groups and *in-vitro* validation techniques.

### Future Work and Potential Applications

As mentioned before, RPTS was engineered to support the analysis of 3’ and 5’-UTR arrays as a function of RBP affinity. It is our ultimate goal to expand the basic theories and function of RPTS to support a multitude of high-throughput binding data, and expand the downstream analysis of RPTS predictions to screen for interactions with other potent cellular biomolecules such as miRNA.

Nearly all RBP-RNA interactions that are known involve the potential for disruption of the complex through cellular signaling and other biochemical events. If the complex is disrupted, it leaves a consensus motif that is normally bound by RBPs open and a potential target for other effectors to modulate gene stability, silencing, or localization. A future goal of our lab is to create and annotate a database for confirmed RPTS motif predictions that can be used to scan predicted motifs against a multitude of cellular modulators such as miRNA, and other characterized RBP-domains for which a target sequence is already known.

The loss of interaction to regulatory RBPs can be of drastic consequence to the cell and organism as a whole. Many of these epigenetic events can be the starting point for various forms of cancer and cellular de-differentiation. A large database of SNPs is curated by Entrez (dbSNP) and contains an ever-growing number of SNPs that are of clinical significance. RPTS allows for the precise identification of putative identification of an RBP binding motif, and the subsequent genome-wide identification of genes that contain this motif with corresponding motif locations. The combination of this data can be further analyzed and compared with dbSNP in order to correlate a potential mutation that induces loss of function through the loss of interaction with a regulatory RBP.

The output of RPTS maps consensus motifs to the exact position on a gene, we are currently in the process of writing a computational algorithm that will extract these data and map the motif to individual chromosomes to be more compatible with SNP analysis. Many of the annotated SNPs contained in dbSNP give their position by their location in the chromosome. **Fig 4C** is a frequency count of SNPs located in genes predicted by this algorithm to contain an evolutionary conserved motif in their 3’-UTR, and lose binding to p-hnRNP E1. Counts of SNPs contained per gene were obtained for each regulon gene for humans, these values are significantly higher when organismal constraints are removed. From a statistical standpoint, there is a high probability that some of these annotated SNPs will reside in the consensus motif, and depending on the type of SNP, may potentially cause a disruption of interaction with the regulatory RBP. Previous data from our lab shows a single point mutation contained in the regulatory consensus of Dab2 causes a complete loss of binding and regulation [11].

In summary, we have created a unique computational algorithm that is capable of providing a simplistic user interface where a user can simply upload a standardized GEO expression set and output a putative consensus motif descriptor along with a transcriptome wide identification of motif-containing genes.

## Supporting Information

S1 Fig Additional RBPs analyzed by RPTS. We have included data for two additional RBPs from high-throughput arrays, Nova2, and Tdp43. Lists contain predicted genes that have a unique regulatory motif for Nova2 and Tdp43, lists were generated by setting a 3-fold enrichment constraint and a minimum of 4-orthologous genes.
(TIFF)

## References

[1] Kuersten S, Goodwin EB. The power of the 3[prime] UTR: translational control and development. Nat Rev Genet. 2003;4(8):626–37. 12897774 10.1038/nrg1125

[2] Aletta JM, Cimato TR, Ettinger MJ. Protein methylation: a signal event in post-translational modification. Trends in Biochemical Sciences. 1998;23(3):89–91. 10.1016/S0968-0004(98)01185-2. 9581497

[3] Dreyfuss G, Kim VN, Kataoka N. Messenger-RNA-binding proteins and the messages they carry. Nat Rev Mol Cell Biol. 2002;3(3):195–205. 11994740 10.1038/nrm760

[4] Ray D, Kazan H, Cook KB, Weirauch MT, Najafabadi HS, Li X, et al A compendium of RNA-binding motifs for decoding gene regulation. Nature. 2013;499(7457):172–7. 10.1038/nature12311 23846655 PMC3929597

[5] Chen K, Rajewsky N, editors. Deep conservation of microRNA-target relationships and 3'UTR motifs in vertebrates, flies, and nematodes Cold Spring Harbor symposia on quantitative biology; 2006: Cold Spring Harbor Laboratory Press. 10.1101/sqb.2006.71.039 17381291

[6] Siomi H, Matunis MJ, Michael WM, Dreyfuss G. The pre-mRNA binding K protein contains a novel evolutionary conserved motif. Nucleic acids research. 1993;21(5):1193–8. 8464704 10.1093/nar/21.5.1193 PMC309281

[7] Wuchty S, Oltvai ZN, Barabasi AL. Evolutionary conservation of motif constituents in the yeast protein interaction network. Nat Genet. 2003;35(2):176–9. 10.1038/ng1242 . 12973352

[8] Churbanov A, Rogozin IB, Babenko VN, Ali H, Koonin EV. Evolutionary conservation suggests a regulatory function of AUG triplets in 5′-UTRs of eukaryotic genes. Nucleic Acids Research. 2005;33(17):5512–20. 10.1093/nar/gki847 16186132 PMC1236974

[9] Mukhopadhyay R, Jia J, Arif A, Ray PS, Fox PL. The GAIT system: a gatekeeper of inflammatory gene expression. Trends Biochem Sci. 2009;34(7):324–31. 10.1016/j.tibs.2009.03.004 19535251 PMC3637685

[10] Maglott D, Ostell J, Pruitt KD, Tatusova T. Entrez Gene: gene-centered information at NCBI. Nucleic acids research. 2011;39(suppl 1):D52–D7. 21115458 10.1093/nar/gkq1237 PMC3013746

[11] Chaudhury A, Hussey GS, Ray PS, Jin G, Fox PL, Howe PH. TGF-[beta]-mediated phosphorylation of hnRNP E1 induces EMT via transcript-selective translational induction of Dab2 and ILEI. Nat Cell Biol. 2010;12(3):286–93. http://www.nature.com/ncb/journal/v12/n3/suppinfo/ncb2029_S1.html. 10.1038/ncb2029 20154680 PMC2830561

[12] Hussey GS, Chaudhury A, Dawson AE, Lindner DJ, Knudsen CR, Wilce MC, et al Identification of an mRNP complex regulating tumorigenesis at the translational elongation step. Molecular cell. 2011;41(4):419–31. 10.1016/j.molcel.2011.02.003 21329880 PMC3061437

[13] Hussey GS, Link LA, Brown AS, Howley BV, Chaudhury A, Howe PH. Establishment of a TGFβ-induced post-transcriptional EMT gene signature. PloS one. 2012;7(12):e52624 10.1371/journal.pone.0052624 23285117 PMC3527574

[14] Pesole G, Liuni S, Grillo G, Licciulli F, Larizza A, Makalowski W, et al UTRdb and UTRsite: specialized databases of sequences and functional elements of 5′ and 3′ untranslated regions of eukaryotic mRNAs. Nucleic Acids Research. 2000;28(1):193–6. 10592223 10.1093/nar/28.1.193 PMC102415

[15] Pesole G, Liuni S, Grillo G, Licciulli F, Mignone F, Gissi C, et al UTRdb and UTRsite: specialized databases of sequences and functional elements of 5′ and 3′ untranslated regions of eukaryotic mRNAs. Update 2002. Nucleic acids research. 2002;30(1):335–40. 11752330 10.1093/nar/30.1.335 PMC99102

[16] Clamp M, Cuff J, Searle SM, Barton GJ. The jalview java alignment editor. Bioinformatics. 2004;20(3):426–7. 14960472 10.1093/bioinformatics/btg430

[17] Edgar RC. MUSCLE: multiple sequence alignment with high accuracy and high throughput. Nucleic acids research. 2004;32(5):1792–7. 15034147 10.1093/nar/gkh340 PMC390337

[18] Thompson JD, Muller A, Waterhouse A, Procter J, Barton GJ, Plewniak F, et al MACSIMS: multiple alignment of complete sequences information management system. BMC bioinformatics. 2006;7(1):318. 16792820 10.1186/1471-2105-7-318 PMC1539025

[19] Goujon M, McWilliam H, Li W, Valentin F, Squizzato S, Paern J, et al A new bioinformatics analysis tools framework at EMBL–EBI. Nucleic acids research. 2010;38(suppl 2):W695–W9. 20439314 10.1093/nar/gkq313 PMC2896090

[20] Sievers F, Wilm A, Dineen D, Gibson TJ, Karplus K, Li W, et al Fast, scalable generation of high‐quality protein multiple sequence alignments using Clustal Omega. Molecular systems biology. 2011;7(1):539. 21988835 10.1038/msb.2011.75 PMC3261699

[21] Boratyn GM, Camacho C, Cooper PS, Coulouris G, Fong A, Ma N, et al BLAST: a more efficient report with usability improvements. Nucleic acids research. 2013;41(W1):W29–W33. 23609542 10.1093/nar/gkt282 PMC3692093

[22] Mootha VK, Lindgren CM, Eriksson K-F, Subramanian A, Sihag S, Lehar J, et al PGC-1α-responsive genes involved in oxidative phosphorylation are coordinately downregulated in human diabetes. Nature genetics. 2003;34(3):267–73. 12808457 10.1038/ng1180

[23] Subramanian A, Tamayo P, Mootha VK, Mukherjee S, Ebert BL, Gillette MA, et al Gene set enrichment analysis: a knowledge-based approach for interpreting genome-wide expression profiles. Proceedings of the National Academy of Sciences of the United States of America. 2005;102(43):15545–50. 16199517 10.1073/pnas.0506580102 PMC1239896

[24] Huo L-R, Ju W, Yan M, Zou J-H, Yan W, He B, et al Identification of differentially expressed transcripts and translatants targeted by knock-down of endogenous PCBP1. Biochimica et Biophysica Acta (BBA)-Proteins and Proteomics. 2010;1804(10):1954–64. 20624489 10.1016/j.bbapap.2010.07.002

[25] Shi Z, Zhang T, Long W, Wang X, Zhang X, Ling X, et al Down-regulation of poly (rC)-binding protein 1 correlates with the malignant transformation of hydatidiform moles. International Journal of Gynecological Cancer. 2012;22(7):1125–9. 10.1097/IGC.0b013e3182606ac3 22801034

[26] Xue X, Wang X, Liu Y, Teng G, Wang Y, Zang X, et al SchA–p85–FAK complex dictates isoform-specific activation of Akt2 and subsequent PCBP1-mediated post-transcriptional regulation of TGFβ-mediated epithelial to mesenchymal transition in human lung cancer cell line A549. Tumor Biology. 2014;35(8):7853–9. 10.1007/s13277-014-1982-1 24819169

