# Supplementary figures and images for: Computational Identification of Post Translational Modification Regulated RNA Binding Protein Motifs
Source: PLoS One. 2015 Sep 14;10(9):e0137696. doi: 10.1371/journal.pone.0137696 (PMC4569568; doi:10.1371/journal.pone.0137696)

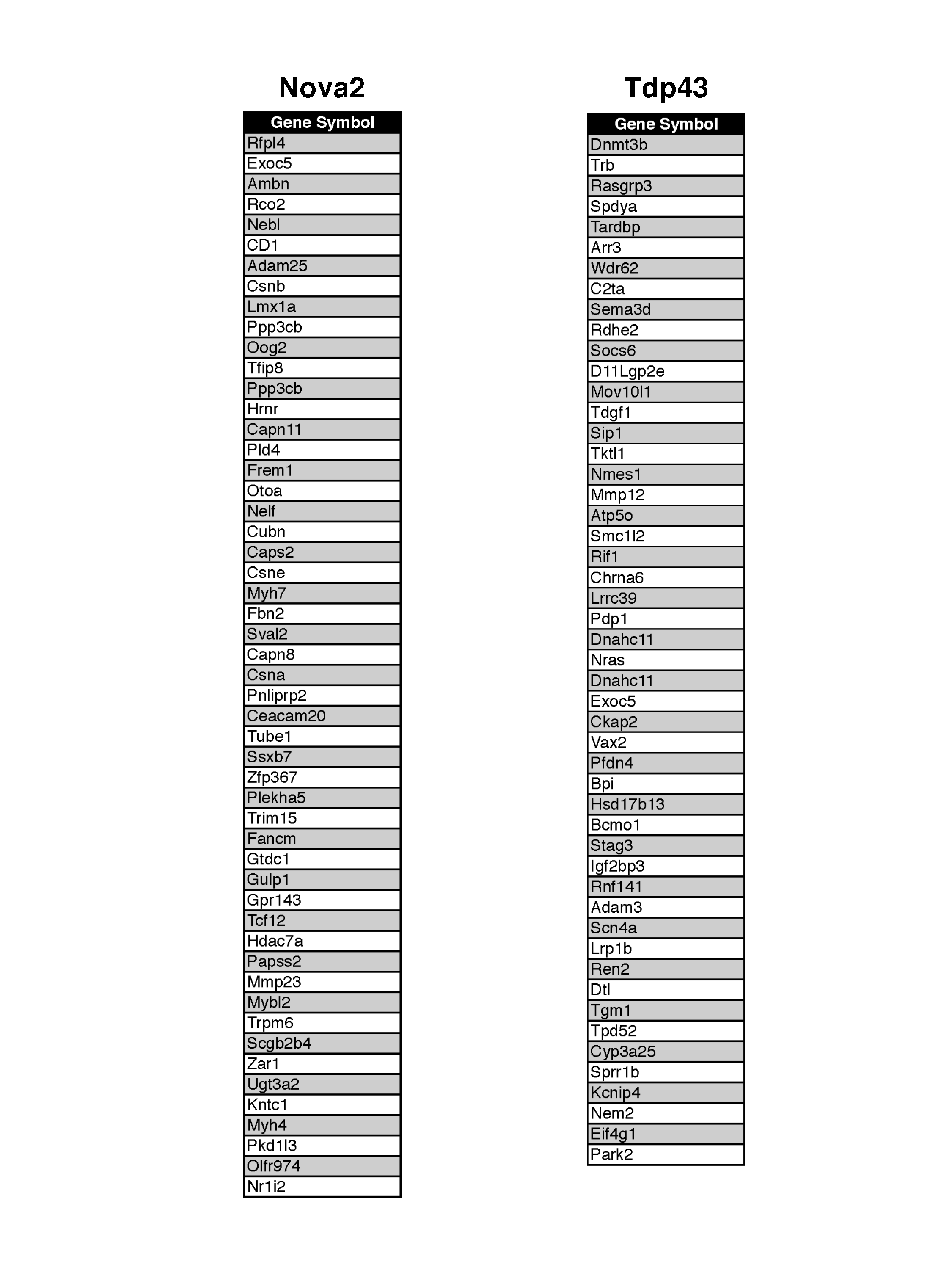

Supplement: S1 Fig — We have included data for two additional RBPs from high-throughput arrays, Nova2, and Tdp43. Lists contain predicted genes that have a unique regulatory motif for Nova2 and Tdp43, lists were generated by setting a 3-fold enrichment constraint and a minimum of 4-orthologous genes. (TIFF) [file pone.0137696.s001.tiff]
